# Designing ultrathin film composite membranes: the impact of a gutter layer

**DOI:** 10.1038/srep15016

**Published:** 2015-10-12

**Authors:** Moon Kattula, Koushik Ponnuru, Lingxiang Zhu, Weiguang Jia, Haiqing Lin, Edward P. Furlani

**Affiliations:** 1 Department of Chemical and Biological Engineering, University at Buffalo, The State University of New York, Buffalo, NY 14260, USA; 2 Department of Electrical Engineering, University at Buffalo, The State University of New York, Buffalo, NY 14260, USA

## Abstract

Industrial membranes comprised of a thin selective layer (<100 nm) requires a gutter layer (<100 nm) between the selective layer and the porous support to achieve high permeance for gas separation. The gutter layer materials must be carefully chosen to enhance overall membrane performance, *i.e.*, high permeance and high selectivity. However, the experimental determination of the optimum gutter layer properties is very challenging. Herein we address this need using a three dimensional (3D) computational model to systematically determine the effects of the gutter layer thickness and permeability on membrane performance. A key finding is that the introduction of a gutter layer between the selective layer and porous support can enhance the overall permeance of the penetrant by up to an order of magnitude, but this gain is accompanied by an undesired decrease in selectivity. The analysis also shows for the first time that a maximum increase in permeance with negligible decrease in selectivity is realized when the thickness of the gutter layer is 1-2 times the pore radius. The modeling approach provides clear and practical guidelines for designing ultrathin multilayer composite membranes to achieve high permeance and selectivity for low-cost and energy-efficient molecular separations.

Membrane technology has been widely used for water purification and gas separation, due to its inherent advantages over conventional separation technologies, such as high energy efficiency, simplicity in operation, compactness and ease of scale-up^1^,^2^,^3^,^4^. Membrane processes have emerged as the leading technology for seawater desalination, nitrogen enrichment from air, and CO_2_ removal from natural gas^1^. Membranes also provide an attractive alternative for emerging applications such as CO_2_ capture from coal power plant-derived flue gas and syngas^5^,^6^,^7^, and water reuse from hydraulic fracking-produced water^8^. The key to the success of membrane technology is ultrathin film composite membranes with high permeance, resulting in the low capital cost for membrane systems. However, despite of their commercial importance, relatively few theoretical studies have been reported that focus on the rational design of ultrathin film composite membranes. In this paper we demonstrate a 3D modeling approach that provides fundamental understanding, and enables the design and optimization of multilayered ultrathin composite membrane structures that achieve high permeance and selectivity.

Figure 1a shows a schematic of a conventional two layer composite membrane for gas separation^1^. The thin dense polymer layer (<100 nm) performs molecular separation while the bulk porous support layer (150–200 μm) provides mechanical strength with negligible mass transport resistance. The support typically has low porosity ranging from 0.01 to 0.1 and small pores (<100 nm) on the surface to provide a relatively smooth surface for the deposition of the selective layer^1^,^9^,^10^,^11^. The characteristic flux of the membrane for gas A, *i.e.*, the permeance P_*A*_, is defined as^12^:


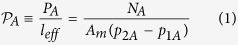


where *P*_*A*_ is the permeability of penetrant A in the selective layer, *l*_*eff*_ is the effective diffusion length of the penetrant, *N*_*A*_ is the steady-state flux of penetrant A through the membrane, *A*_*m*_ is the membrane surface area, and *p*_*2A*_ and *p*_*1A*_ are the upstream (high) and downstream (low) partial pressure of A, respectively. In general, the effective diffusion length *l*_*eff*_ is greater than the geometric layer thickness *l*_*s*_ due to the bending of the concentration streamlines as shown below. The selectivity of component A over B (*α*_*A*/*B*_) is defined as the ratio of their permeance values, *i.e.*, α_A/B_ = 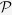
_A_/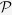
_B._

Membranes should have high selectivity to achieve the required product purity, and high permeance to reduce the required membrane area that often scales linearly with the capital cost of the membrane system^4^,^5^. As indicated by Eq. 1, the permeance can be enhanced by molecularly designing selective layer materials to achieve superior permeability^13^,^14^,^15^,^16^,^17^, or by decreasing *l*_*eff*_. At first glance, it appears that the most straightforward way to increase the permeance is to reduce *l*_*eff*_, by reducing the selective layer thickness *l*_s_^18^,^19^,^20^,^21^,^22^. However, as the thickness of the selective layer is reduced and becomes comparable to the surface pore size of the porous support, the surface morphology of the support (porosity and pore size) geometrically restricts penetrant diffusion in the selective layer, which greatly reduces permeance^10^,^18^,^19^. Specifically, the geometric restriction increases the effective diffusion length *l*_*eff*_ for the penetrant as indicated by the curved red arrows shown in Fig. 1a. This leads to a non-linear penetrant concentration profile and a corresponding reduction in flux *N*_*A*_, and thus permeance^18^,^20^. The effect of the support surface morphology on penetrant permeance has been rationalized to some extent using both analytical models^19^,^21^,^22^,^23^,^24^,^25^, and numerical modeling that describes the concentration profile and flux within the selective layer^10^,^25^,^26^. However, this prior work was limited to conventional two layer membrane structures as shown in Fig. 1a.

To mitigate the aforementioned geometric restriction due to the porous support, three layer structures are used wherein a highly permeable “gutter” layer is introduced between the selective layer and the porous support as shown in Fig. 1b^19^,^27^,^28^. The gutter layer is often prepared from materials with extremely high permeability but low selectivity, such as poly[(1-trimethylsilyl)-1-propyne] (PTMSP)^29^ and polydimethylsiloxane (PDMS)^30^. Due to its high permeability, the gutter layer channels the permeate into the surface pores, thereby reducing the geometric restriction characteristic of two layer structures, without adding significant transport resistance^19^. The effect of the gutter layer on the membrane permeance has been described using a 2D analytical model^19^,^21^. However, this model does not provide a rigorous understanding of membrane behavior and does not accurately predict the penetrant concentration profile in the membrane. Thus, a need exists for a more rigorous model that addresses these deficiencies.

In this paper we demonstrate a 3D computational model that provides a precise understanding of the effect of the membrane nano-features on its separation performance. The model predicts the penetrant concentration profile and flux in multilayer ultrathin membrane structures (as shown in Fig. 1b) as a function of the constituent material dimensions and properties^10^,^26^,^31^,^32^,^33^,^34^. A key finding of this work is that the gutter layer can enhance the overall permeance of the penetrant by up to an order of magnitude, but this gain is accompanied by an undesired decrease in selectivity. We also show for the first time that optimum membrane performance (*i.e.*, a maximum increase in permeance with negligible decrease in selectivity) is realized when the thickness of the gutter layer is in the range of 1–2 times the pore radius. The modeling approach is very general and should be of considerable use in designing ultrathin film composite membranes to achieve high gas permeance and selectivity.

## Methods

**Figure 1:**
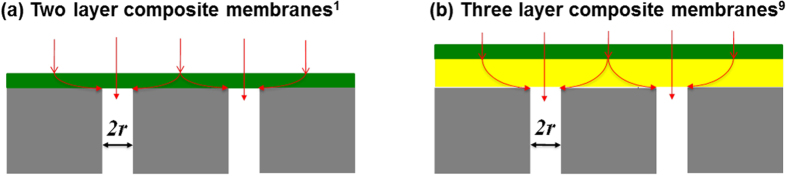
Schematics of ultrathin film composite membranes: (a) a conventional two-layer membrane comprised of a selective layer (with thickness *l*_*s*_) on top of a porous support; (b) a three-layer composite membrane with a gutter layer of a thickness of *l*_*g*_ between the selective layer and support.The support has a pore radius of *r*.

**Figure 2:**
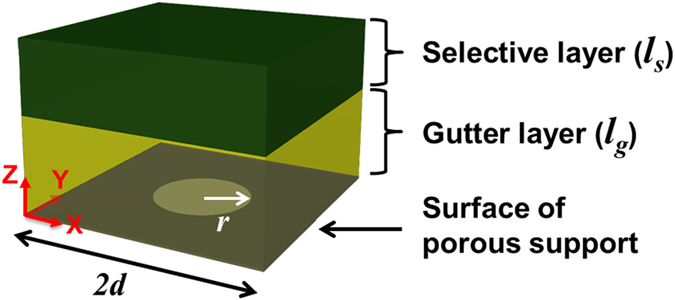
Schematic of a cubic unit cell and associated dimensions for computational modeling. *r*: pore radius of the porous support; *2d*: unit cell length; porosity 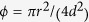; scaled selective layer thickness, 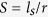; and scaled gutter layer thickness, 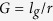.

We use a 3D computational model to predict steady-state penetrant transport in composite membranes and the impact of membrane nano-features on separation performance. Two- and three—layer membranes are considered as shown in Fig. 1. It is assumed that there is no pore penetration of the selective layer or gutter layer material, and these materials have permeability values independent of film thickness. By assuming a 2D array of uniformly spaced cylindrical pores in the porous support, we can exploit the symmetry of this ordered pore structure and reduce the analysis to a unit cell of the membrane as shown in Fig. 2. Symmetry boundary conditions are applied on the sides of the unit cell to account for the surrounding membrane structure. The penetrant transport in the selective and gutter layers is driven by the concentration gradient following the solution -diffusion model^12^. The equation that governs the steady-state concentration (*C*_*A*_) of a penetrant A in the membrane is given below^10^:


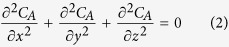


To simplify the analysis, the concentration *C*_*A*_ is set as 1 at the surface of the selective layer exposed to the feed gas, and 0 at the bottom of the gutter layer exposed to the permeate gas. The latter boundary condition (*C*_*A*_ = 0) implies that transport resistance through the pore is much less than through the selective and gutter layers, and thus the penetrant can be considered to be instantaneously removed at the gutter layer-permeate interface. Based on these boundary conditions, the predicted penetrant concentration in the selective and gutter layers will be between 0 to 1, which is convenient for modeling without affecting the calculation of gas permeance^10^.

The steady-state transport of penetrant A is obtained by solving Eq. 2 subject to appropriate boundary conditions. The penetrant concentration is assumed to be continuous at the interface between the selective and gutter layers for the simplicity of analysis^10^. At steady state, the flux is constant in the selective and gutter layers:


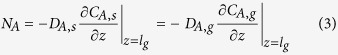


where 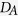 is the diffusion coefficient for penetrant A, and the subscripts of *s* and *g* indicate the selective and gutter layers, respectively. The nonporous region of the porous support is assumed to be impermeable and there is also zero-flux of the penetrant in the *x* and *y* direction through the sides of the unit cell because symmetry conditions are imposed at these boundaries.

The numerical model was implemented in the COMSOL multiphysics program (www.comsol.com), which solves the governing differential equations using the finite element method (FEM). In the FEM the equations with appropriate boundary conditions are transformed and solved as a system of algebraic equations by discretizing the computational domain into a mesh of grid points. The accuracy of the solution typically increases as the mesh size decreases. For this analysis, we set the minimum and maximum mesh size to 10^−11^ and 10^−9^ meters, respectively. We found that it was necessary to use adaptive mesh refinement throughout the computational domain to deal with abrupt changes in material properties, which was especially needed at the edge of the pores where the boundary condition abruptly changes from the zero-flux condition in the *z* direction to a constant concentration of zero^10^.

The computational model predicts the concentration profile in the selective and gutter layers, and the resulting flux *N*_*A*_ across the composite membrane. The geometric restriction of the membrane nano-features on the observed permeance of penetrant A can be characterized as membrane permeance efficiency, *β*_*A*_:


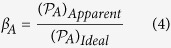


where 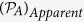 is the membrane permeance modeled and 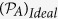 is the ideal permeance of the selective layer without the influence of the gutter layer and porous support (*i.e.*, 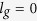 and 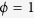). Lower 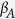 values indicate greater deviation from the ideal permeance, reflecting more severe effect from the gutter layer and/or the porous support on the gas permeation.

## Results and Discussion

### Geometric effect of the porous support on membrane performance

**Figure 3:**
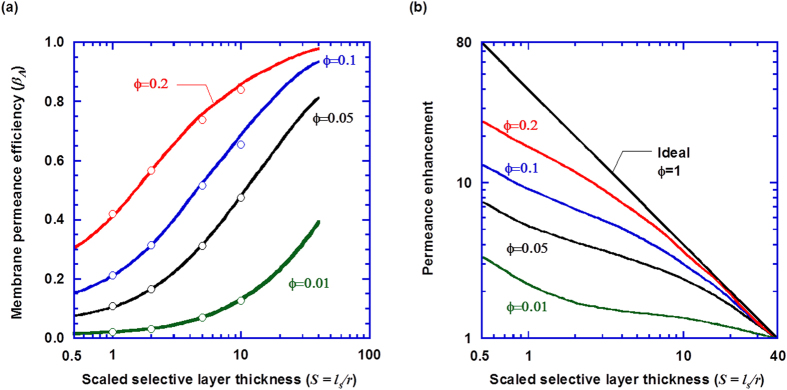
Effect of the support porosity and the scaled selective layer thickness on (a) the membrane permeance efficiency and (b) permeance enhancement (defined as the ratio of apparent permeance at any *S* value to that at *S* = **40) in the two-layer composite membrane shown in** Fig. 1a. The circles in (a) represent data taken from the literature^10^, which is used to validate the computational approach and model presented herein.

We first model a two-layer membrane structure as shown in Fig. 1a to understand the effect of the porous structure on permeance and to validate our prediction using the published data. Figure 3a shows the membrane permeance efficiency *β*_*A*_ as a function of the support porosity and the scaled selective layer thickness (*S* = *l*_*s*_/*r*). The simulation results depend on the porosity, and the scaled selective layer thickness, but not the absolute value of *l*_*s*_ or *r*^10^,^18^. In addition to the typical porosity values ranging from 0.01 to 0.1^10^,^11^, a porosity as high as 0.2 is also investigated in this work to explore the maximal benefits attainable by increasing the porosity. As shown in Fig. 3a, increasing porosity increases the membrane permeance efficiency. At a typical support porosity of 0.05 and a scaled selective layer thickness of 2, the permeance efficiency is as low as 0.17, indicating a significant flux restriction imposed by the porous support. These results are also consistent with those simulated for water permeation by Ramon and coauthors, as represented as circles in Fig. 3a^10^.

Decreasing the selective layer thickness increases the ideal permeance, which, however, decreases the permeance efficiency. Figure 3b directly shows the compromised benefits of decreasing selective layer thickness as a function of porosity (*ϕ*). For example, decreasing the selective layer thickness from *S* = 40 to *S* = 0.5 is expected to increase the ideal permeance by 80 times. However, due to the geometric restriction of the porous support, the permeance enhancement at *S* = 0.5 (defined as the ratio of apparent permeance at *S* = 0.5 value to that at *S* = 40) is only 3.6, 7.6, and 13 at *ϕ* = 0.01, 0.05, and 0.1, respectively. Even for a hypothetical porous support with *ϕ* = 0.2, the expected 80 times increase in the ideal permeance yields only 25 times increase in the apparent permeance.

### Geometric effect of the gutter layer on membrane performance

**Figure 4:**
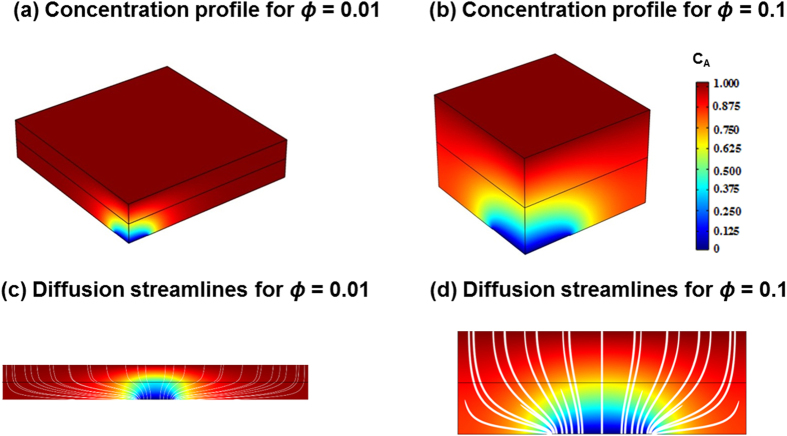
Cutaway view of the 3D model unit cell showing the concentration profile in a quarter of a unit cell for a membrane with a gutter layer at (a) *ϕ *=* *0.01 and (b) *ϕ *=* *0.1; Diffusive streamlines along with the concentration profile shown along *x*-*z* plane with a gutter layer at (c) *ϕ* = 0.01 and (d) *ϕ* = 0.1. In these calculations, *S* = *G* = 1; 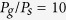.

As illustrated in Fig. 1b, a gutter layer with negligible mass transfer resistance can channel the gas flow and thus mitigate geometric restriction by the porous support. Figures 4a,b depict the magnitude of the 3D concentration of the penetrant for the three-layer membranes comprised of porous supports of *ϕ* = 0.01 and *ϕ* = 0.1, respectively. These two porosity values represent the two extremes for commercially available porous supports^10^,^11^. All of the calculations assume *S* = *G* = 1 and 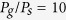. Figures 4a,b provide a clear visualization of a significant concentration gradient near the pores, which indicates that the pores impose a substantial resistance to mass transport. Increasing porosity mitigates the pore restriction. In Fig. 4c,d, the 3D concentration streamlines are shown along with the concentration profile which represent diffusive paths through the selective and gutter layers. The pores increase the effective path length *l*_*eff*_ of molecular transport and hence decrease the permeance. The increase in the path length is diminished with increasing porosity. Since the restriction is near the pore regions, increasing the thickness of the selective layer and/or the gutter layer would minimize the pore restriction, and increase the concentration distribution in these layers.

Figures 4c,d also illustrates the benefits of using a gutter layer. With the gutter layer, the diffusion streamlines are almost parrallel to the gas permeation direction in the selective layer where the major transport resistance lies, while the bending of the diffusion streamline is mainly in the gutter layer. On the other hand, if there was no gutter layer, the streamline in the selective layer would be almost the same as that in the gutter layer, which is much longer than the film thickness due to the restriction from the porous support.

**Figure 5:**
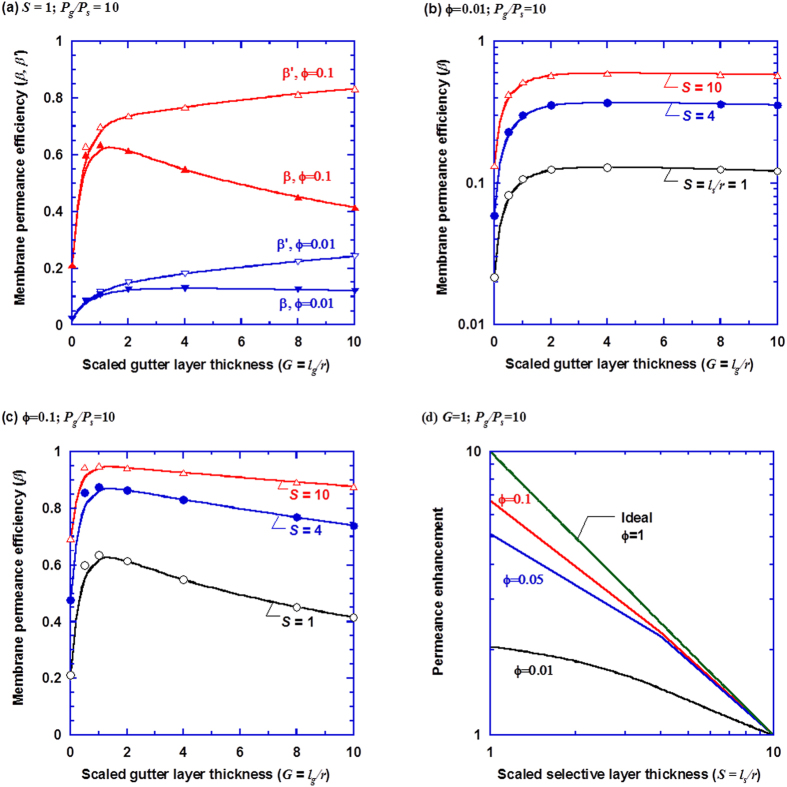
Effect of the scaled gutter layer thickness(*G* = *l*_g_*/r*) on (a) membrane permeance efficiency (β_A_) and 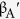 (defined as the ratio of the apparent permeance to the combined selective and gutter layer); (b) ϕ = 0.01; (c) ϕ = 0.1; and (d) permeance enhancement by decreasing selective layer thickness at G = 1 and various porosities. In all simulations, the permeability of the gutter layer is ten times that of the selective layer.

Figure 5 illustrates the effect of the gutter layer on membrane permeance efficiency for porous supports with two extreme porosities (*i.e., ϕ* = 0.01 and 0.1). In this analysis the gutter layer is assumed to have 10 times the permeability of the selective layer. The introduction of a gutter layer significantly increases permeance efficiency, though adding the gutter layer is expected to increase the mass transport resistance. As shown in the curves for membrane permeance efficiency (*β*_*A*_) in Fig. 5a, introducing a thin gutter layer can increase the *β*_*A*_ value from 0.022 to as high as 0.13 (a 5.9-fold increase) at *G* = 4 and *ϕ* = 0.01, and from 0.21 to 0.64 (a 3.0-fold increase) at *G* = 1 and *ϕ* = 0.1.

Figure 5a also shows the membrane permeance efficiency for overall selective and gutter layers (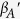), which is defined as the apparent permeance to the ideal permeance of the combined selective and gutter layer (with a total length of *l*_*s*_ + *l*_*g*_),


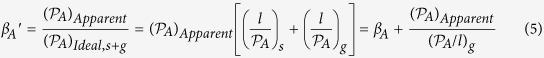


where the subscripts of *s, g, s* + *g* indicate the permeances for the selective layer only, gutter layer only, and the combined selective and gutter layers, respectively. Clearly, increasing the gutter layer thickness reduces the geometric restriction and thereby increases the 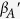 values. On the other hand, a further increase in the gutter layer thickness increases mass transport resistance in the gutter layer, which decreases the membrane permeance. Therefore, the benefit of reduced geometric restriction by the thicker gutter layer can be diminished by the increased mass transport resistance. For example, as the scaled gutter layer thickness increases to above 2, there is no benefit for the membrane permeance efficiency (*β*_*A*_) for the porous support of *ϕ* = 0.01, and it even decreases the permeance efficiency (*β*_*A*_) for *ϕ* = 0.1, as shown in Fig. 5a. There needs a judicious choice for the gutter layer material with high permeability and a balanced thickness to achieve the maximal improvement of membrane permeance.

Figures 5b,c show the quantitative effect of the gutter layer on membrane permeance efficiency at a scaled selective layer thicknesses ranging from 1 to 10, and porosity of 0.01 and 0.1. For these conditions, there seems to be an optimal scaled gutter layer thickness (*G*) between 1 and 2 to achieve the highest increase in permeance efficiency for various *S* values. The observation is also valid for the cases of *P*_*G*_*/P*_*S*_ = 5. Increasing the selective layer thickness increases the permeance efficiency, which is consistent to the study of two-layer composite membranes, as shown in Fig. 3. The most benefit of introducing the gutter layer is observed for the membranes with low porosity and thin selective layer, where the geometric restriction is most severe.

Figure 5 also illustrates the importance of the porous supports. At *ϕ* = 0.01, the best membrane permeance efficiency that can be achieved is 0.12 for *S* = 1, and 0.58 for *S* = 10. On the other hand, at *ϕ* = 0.1, the greatest permeance efficiency achievable is 0.64 for *S* = 1, and 0.94 for *S* = 10. Therefore, porous supports with finer pores (lower *r* values and hence higher *S* values) and higher porosity are preferred. However, higher porosity may lead to the pore penetration of the coating solution during the membrane preparation, which blocks the pores and increases the support transport resistance, and thus decrease the membrane permeance^19^,^35^. Consequently, most commercial porous supports may have porosity less than 0.1. There remains a great challenge in producing the porous support with the balanced characteristics needed to prepare ultrathin membranes with high flux.

Figure 5d directly exhibits the benefit of decreasing the selective layer thickness at different support porosities. The condition of *G* = 1 is chosen here for illustration, because it provides one of the greatest permeance efficiency values. While decreasing the selective layer thickness from *S* = 10 to *S* = 1 increases the ideal permeance by 10 times, the enhancement in apparent permeance is 2.0 at *ϕ* = 0.01, and 6.7 at *ϕ* = 0.1. To put this in perspective, without the gutter layer, the permeance enhancement is only 1.5 at *ϕ* = 0.01, and 5.1 at *ϕ* = 0.1, when the *S* value decreases from 40 to 4, as shown in Fig. 3b. It should be noted that the permeance efficiency is much lower for the two-layer membranes compared to the three-layer membranes, as shown in Fig. 5a. The results strongly indicate that the porous support and gutter layer are critical to designing high flux ultrathin membranes.

### Selection of gutter layer materials

**Figure 6:**
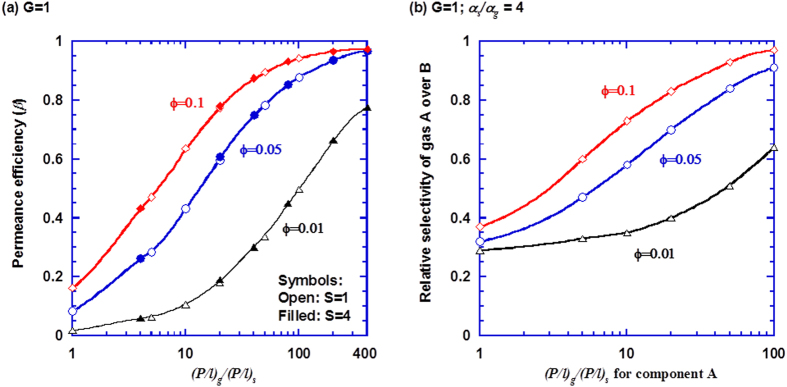
Effect of the support porosity (*ϕ*) and the permeance ratio of the gutter layer to selective layer (*i.e.*, 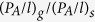) on (a) the permeance efficiency (*β*_*A*_); and (b) the relative gas selectivity (defined as the ratio of apparent selectivity of gas A over B to ideal selectivity). The points are simulated data and the curves are to guide the eye.

To provide a quantitative guidance of selecting the gutter layer materials, we performed simulations using a gutter layer with various permeability values. The scaled gutter layer thickness (*G*) is set at 1 for all simulations, because it yields the highest permeance efficiency among those considered. Figure 6a shows the effect of the ideal permeance ratio of the gutter layer to the selective layer (*i.e.*, 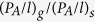) on the membrane permeance efficiency. The permeance efficiency increases with increasing the support porosity and 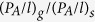 values.

At the constant *G* of 1, the permeance efficiency depends on the value of 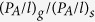, but not on the scaled selective layer thickness, presumably because the mass transfer resistance (or the permeance) is the critical factor, instead of the individual parameter of permeability or thickness. The trend is consistent to the conventional notion that the preferred gutter layer should have much higher permeance than the selective layer. In the practical range of 10–100 for 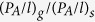, the permeance efficiency is still low for the porosity of 0.01 and 0.05, which suggests that membranes with high flux should be fabricated on a porous support with porosity of 0.1 or above. For example, at *ϕ* = 0.1, the permeance efficiency is as high as 0.80 with a moderate value of 20 for 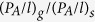.

Membrane materials with higher permeability are expected to have lower selectivity, *i.e.*, the gutter layer has higher permeability and lower selectivity (*α*_*A*/*B*_) than the selective layer^36^,^37^,^38^. Consequently, the more permeable component (A) has a lower value of 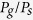 and thus a lower value of permeance efficiency (*β*_*A*_), compared to the less permeable component (*β*_*B*_). The apparent selectivity of component A over B in membranes, 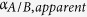, can be expressed as:


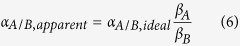


where *α*_*A*/*B*_,_*ideal*_ is the selectivity without the effect of the gutter layer and porous support (*i.e.*, at *l*_*s*_ = 0 and 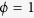). Since 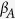 is lower than 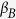, the apparent selectivity is often lower than the ideal selectivity, which is captured in Fig. 6b. In the simulations, the selectivity in the selective layer is set to be 4 times of the gutter layer (*i.e.*, 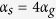). The gutter layer permeability for the more permeable component A has significant impact on the apparent selectivity. As the value of 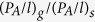 increases from 1 to 100, the relative selectivity (defined as the ratio of the apparent selectivity to ideal selectivity) increases from 0.32 to 0.91 at 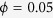. More specifically, considering a case with *S* = 1 and 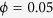, the permeance efficiency is as low as 0.10 without the gutter layer. Introducing a gutter layer with *G* = 1 and 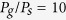 for component A would increase the apparent permeance by 4.2 times for component A, and 7.2 times for component B (with 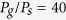), resulting in 42% reduction in the apparent selectivity. The adverse effect of the gutter layer on the selectivity can be minimized by selecting more permeable and selective gutter layer materials and porous support with higher porosity. It is worth noticting that the gutter layer materials should also have good compatibility with the selective layer for the successful deposition of the defect-free thin selective layer.

## Conclusions

Our findings suggest that a thin gutter layer is needed to achieve high permeance in designing ultrathin composite membranes for gas separation. However, the gutter layer can decrease the gas selectivity when the porous support has low porosity. More specifically, a gutter layer thickness of 1–2 times of the pore radius of the porous support yields the maximum improvement in the membrane permeance without significantly decreasing its selectivity, when the relative permeability of the gutter layer to the selective layer is 5–10. The permeance efficiency also increases with increasing porosity and decreasing pore size for the porous supports. The 3D computational model presented herein is readily implemented in commercially available software and should find widspread use in the rational design and optimization of ultrathin multilayer composite membranes.

## Additional Information

**How to cite this article**: Kattula, M. *et al.* Designing ultrathin film composite membranes: the impact of a gutter layer. *Sci. Rep.* **5**, 15016; doi: 10.1038/srep15016 (2015).
